# Temporal trend analysis of hospitalizations and in-hospital deaths due to female breast cancer in the state of Alagoas from 2009 to 2019: a cross-sectional study

**DOI:** 10.1590/1516-3180.2021.0385.R1.23072021

**Published:** 2022-03-14

**Authors:** Myra Jurema da Rocha Leão, Vitória Ingryd dos Santos Cardoso, Ayara Jhulia Palmeira Dantas Lima, Samilla Cristinny Santos, Emmylly Maria Correia Ferro de Araújo, Karen da Costa Paixão, Lucas Gomes Santos, Carlos Alberto de Carvalho Fraga, Carlos Dornels Freire de Souza, Carolinne de Sales Marques

**Affiliations:** I MD. Physician and Master’s Student, Department of Oncological Surgery, Santa Casa de Misericórdia de Maceió, Universidade Federal de Alagoas (UFAL), Arapiraca (AL), Brazil.; II Medical Student, Center for Medical Sciences and Nursing, Universidade Federal de Alagoas (UFAL), Arapiraca (AL), Brazil.; III Medical Student, Center for Medical Sciences and Nursing, Universidade Federal de Alagoas (UFAL), Arapiraca (AL), Brazil.; IV Medical Student, Center for Medical Sciences and Nursing, Universidade Federal de Alagoas (UFAL), Arapiraca (AL), Brazil.; V Medical Student, Center for Medical Sciences and Nursing, Universidade Federal de Alagoas (UFAL), Arapiraca (AL), Brazil.; VI Medical Student, Center for Medical Sciences and Nursing, Universidade Federal de Alagoas (UFAL), Arapiraca (AL), Brazil.; VII Medical Student, Center for Medical Sciences and Nursing, Universidade Federal de Alagoas (UFAL), Arapiraca (AL), Brazil.; VIII PhD. Biologist and Professor, Center for Medical Sciences and Nursing, Universidade Federal de Alagoas (UFAL), Arapiraca (AL), Brazil.; IX PhD. Physical Therapist and Professor, Center for Medical Sciences and Nursing, Universidade Federal de Alagoas (UFAL), Arapiraca (AL), Brazil.; X PhD. Biochemistry and Professor, Center for Medical Sciences and Nursing, Universidade Federal de Alagoas (UFAL), Arapiraca (AL), Brazil.

**Keywords:** Breast neoplasms, Epidemiology, Hospitalization, Public health, Health, Breast cancer, Analysis

## Abstract

**BACKGROUND:**

Breast cancer is a common neoplasm in women worldwide. Its varying patterns of incidence and clinical prognosis in Brazil make it an important and complex public health problem that needs to be solved.

**OBJECTIVES:**

To analyze the temporal dynamics of hospital admissions and deaths due to female breast cancer in the state of Alagoas, Brazil, from 2009 to 2019.

**DESIGN AND SETTING:**

Cross-sectional study including secondary data from hospital admissions and deaths due to female breast cancer in Alagoas.

**METHODS:**

A joinpoint regression model was constructed for temporal analysis of hospital admissions and deaths due to female breast cancer in Alagoas, over this period. The hospital information system of the Department of Informatics of the National Health System was used.

**RESULTS:**

There were 5,801 hospitalizations and 633 hospital deaths due to neoplasm in Alagoas over the period. The age group from 50 to 59 years old stood out, corresponding to 28.1% of hospitalizations and 31.1% of registered deaths. An increasing trend in the rate of hospital admissions was observed (average annual percentage change, AAPC = 14.0; P-value < 0.001), from 14.9/100,000 inhabitants in 2009 to 53.6 in 2019. There was a growth trend in the in-hospital mortality rate (AAPC = 19.8; P-value < 0.001), from 6.3% in 2009 to 11.0% in 2019.

**CONCLUSION:**

The results indicated an increasing trend of hospital admissions and mortality rates in the state of Alagoas, with a higher percentage of hospitalizations and deaths in the 50-59 age group.

## INTRODUCTION

Breast cancer is the most common malignant neoplasm among women in most parts of the world. In 2018, the estimated number of new cases diagnosed was 2.1 million, and there were 627,000 deaths due to this disease. In this context, Latin America and the Caribbean are gaining prominence among the places with the highest average incidence rates, with rates of 40 cases per 100,000 women.^1^

A similar scenario is observed in Brazil, in which approximately 59,700 new cases of female breast cancer were registered in 2018. This corresponds to an incidence rate of 56.3 cases per 100,000 women. Disregarding non-melanoma skin tumors, breast cancer corresponds to 29.5% of all malignant tumors estimated for Brazilian women.^2^ For this reason, breast cancer is an important and complex public health problem that needs to be solved, given its varying patterns of incidence and clinical evolution.^3,4^

In Brazil, there are differences in the incidence of breast cancer between the country’s geographical regions: 73.1/100,000 in the south, 69.5/100,000 in the southeast, 52.0/100,000 in the center-west, 40.4/100,000 in the northeast and 19.2/100,000 in the north.^3^ It is likely that the higher rates that are observed in the southeastern and southern regions may be influenced by the higher levels of oncological care infrastructure and disease diagnosis rates in these regions, considering that they have 66% of the chemotherapy rooms and 72% of the radiotherapy equipment.^5^ In addition, risk factors such as smoking are seen more frequently in the populations of these regions.^6^

Although the northeastern region ranks fourth in incidence rates, the projections point to a higher growth rate than in other regions.^5^ It is the second region in absolute numbers of hospital admissions for female breast cancer, with 14,512 cases (21.3%) in 2019, and has the same position in relation to the number of deaths due to female breast cancer, accounting for 3,807 deaths (21.6%) in 2018.^5,7^ In 2020, 13,190 new cases of breast cancer are expected in the northeast, with an estimated risk of 44.29 cases per 100,000 women.^8^

In the state of Alagoas, there were 620 new cases of breast cancer among women in the year 2020, with a crude incidence rate of 35.2 per 100,000 inhabitants.^9^ According to projections and indicators for 2030, among the states of the northeastern region, Alagoas is expected to present the fourth largest growth in the mortality rate and to have the third largest increase in this rate.^10^

## OBJECTIVE

The aim of this study was to analyze the dynamics of hospital admissions and deaths due to female breast cancer in the state of Alagoas over the period from 2009 to 2019.

## METHODS

### Study design, population and period

This was a cross-sectional study on hospital admissions and deaths due to malignant female breast cancer in the state of Alagoas, over the period from 2009 to 2019. In this study, the year 2020 was not included, considering the possible influence of the coronavirus disease-19 (COVID-19) pandemic on the numbers of hospitalizations and deaths.

### Study setting

This study was carried out in the state of Alagoas, which is located in the northeastern region of Brazil. This state had an estimated population of 3.36 million inhabitants in 2020, among whom the female resident population accounted for 51.5% (n = 1,646,684), with the following age distribution: < 20 years (38.2%, n = 629,638); 20-29 years (17.8%, n = 294,415); 30-39 years (14.8%, n = 244,117); 40-49 years (11.5%, n = 189.799); 50-59 years (8.1%, n = 133.660); 60-69 years (2.1%, n = 85.424); 70-79 years (2.8%, n = 46.754); and 80 years and over (1.3%, n = 22,867).¹¹ The state presents a human development index (HDI) of 0.631, which is lower than the national average (HDI 0.699). Its per capita income is R$ 731.0 and 47.2% of the population live in poverty or extreme poverty.^11^

For this study, the 102 municipalities in the state of Alagoas were classified as geographic units for spatial analysis. Among these municipalities that were analyzed, those with the 10 highest rates of hospital admissions and in-hospital mortality were selected to form the results from the present study.

### Variables, data source and data collection

The following variables were evaluated: age group (< 20, 20-29, 30-39, 40-49, 50-59, 60-69, 70-79 and ≥ 80 years); number of hospitalizations; hospital deaths per residence, hospitalization rate per 100,000 inhabitants; and in-hospital mortality rate (%). The following criteria were considered with regard to data collection: Alagoas as a unit of the federation; female gender; periods of hospitalization between 2009 and 2019 and International Classification of Diseases 10^th^ edition (ICD-10) code C50, which refers to malignant breast neoplasm.

Data on hospital admissions and deaths were obtained from the hospital information system of the Department of Informatics of the National Health System (DATASUS). Population data relating to the female population according to municipality were obtained from the Brazilian Institute for Geography and Statistics (Instituto Brasileiro de Geografia e Estatística, IBGE), based on information from the 2010 census and inter-census projections for the other years of the time series. In-hospital mortality data were obtained directly from the DATASUS platform. The following equation was used to calculate the hospitalization rate: [(No. of hospital admissions (2009-2019) due to female breast cancer in the place)/(female population in the place and period)] x 100,000.

### Statistical analyses

A joinpoint regression model was constructed for temporal analysis. This model checks whether a line with several segments (with several joinpoints) is more suitable for explaining the temporal evolution of the data, compared with a straight line or a line with fewer segments.^12^ The trends were classified as stationary, increasing or decreasing, according to the slope of the regression line. In addition, the annual percentage variation (APC) and the average annual percentage variation (AAPC) were calculated, with 95% confidence intervals (95% CI). The statistical significance level was set at 5%. The trends were defined as follows: i) increasing, when the APC or AAPC was significantly positive; ii) decreasing, when the APC or AAPC was significantly negative; or iii) stationary, when there was no significance in these variations. For this analysis, the Joinpoint Regression software, version 4.5.0.1, was used (National Cancer Institute, Bethesda, Maryland, United States). The QGis software, version 2.8.7 (Open Source Geospatial Foundation [OS Geo], Beaverton, Oregon, United States), was used to make choropleth maps for trend analysis.

### Ethical issues

This research was approved by the Research Ethics Committee of the Universidade Federal de Alagoas, through opinion report no. 3.775,018, on December 16, 2019.

## RESULTS

**Figure 1.**
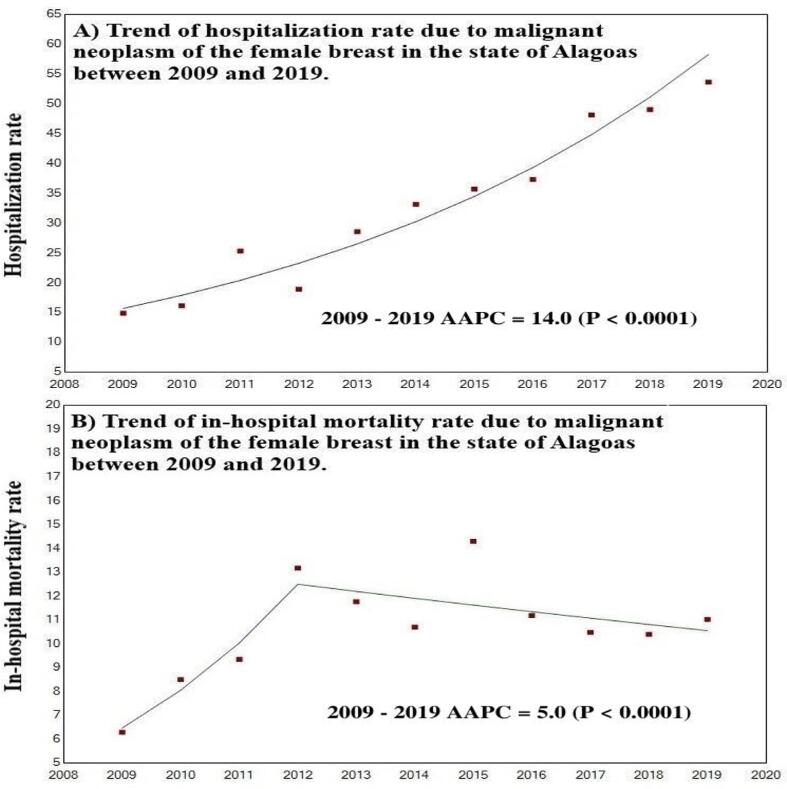
Temporal trend of hospitalization and in-hospital mortality rates due to female breast cancer in the state of Alagoas from 2009 to 2019. Brazil, 2021.

Between 2009 and 2019, 5,801 hospital admissions were reported in the state of Alagoas (accumulated hospitalization rate for the period equal to 35.2/100,000 inhabitants). In the temporal analysis on hospitalization numbers, using the joinpoint regression model, an increasing trend was observed in the state (AAPC = 14.0; 95% CI = 12.3 to 15.8; P-value < 0.001), for which the rate went from 14.9/100,000 inhabitants (n = 239) in 2009 to 53.6 per 100,000 (n = 863) in 2019 (Figure 1A).

A total of 633 hospital deaths were recorded and the in-hospital mortality rate for the period was 11.7%. Regarding the time trend analysis, Alagoas showed a growth trend for female breast cancer (AAPC = 5.0; 95% CI = 0.5 to 9.7; P-value < 0.001), increasing from 6.3% (n = 15) in 2009 to 11.0% (n = 95) in 2019, with temporal inflection in 2012, after which stationary behavior was observed (Figure 1B).

### Analysis on hospital admissions

Regarding the age groups of hospitalizations, 28.1% of the cases (n = 1,627) were among women between 50 and 59 years old, and the lowest percentage was among women under 20 years old (0.8%, n = 47). Among women under 20 years old, there was a declining trend (AAPC = -13.6; 95% CI = - 17.7 to -9.2; P-value < 0.001), whereas for those between 20 and 29 years old there was a tendency towards stability (AAPC = 4.7; 95% CI = - 1.6 to 11.3; p-value = 0.1) and for the other age groups (30-39, 40-49, 50-59, 60-69, 70-79 and ≥ 80 years) there was a growth trend, especially among those between 70 and 79 years old (AAPC = 19.0; 95% CI = 15.4 to 22.8; P-value < 0.001) (Table 1A).

**Table 1.** Temporal trend of hospitalizations due to malignant neoplasm of the female breast in the state of Alagoas and in the 10 municipalities with the highest rates of hospitalizations over the period from 2009 to 2019 and according to the age group. Brazil, 2021

A) Temporal trend of hospitalizations due to malignant neoplasm of the female breast according to age group											
			Trend 1			Trend 2			Total period		
Age group	Number of hospitalizations (%)	Hospitalization rate over the period/100,000 inhabitants	Period	APC	95% CI	Period	APC	95% CI	AAPC	95% CI	P-value
< 20 years	47 (0.8)	0.7	–	–	–	–	–	–	-13.6*	[-17.7 to -9.2]	< 0.001
20-29 years	117 (2.0)	4.0	–	–	–	–	–	–	4.7	[-1.6 to 11.3]	0.1
30-39 years	694 (12.0)	28.4	–	–		–	–	–	11.6*	[7.5 to 15.8]	< 0.001
40-49 years	1509 (26.0)	79.5	–	–	–	–	–	–	14.3*	[11.6 to 17.0]	< 0.001
50-59 years	1627 (28.1)	121.7	–	–	–	–	–	–	15.7*	[13.2 to 18.4]	< 0.001
60-69 years	1062 (18.3)	124.3	–	–	–	–	–	–	13.6*	[11.2 to 16.1]	< 0.001
70-79 years	551 (9.5)	117.8	–	–	–	–	–	–	19.0*	[15.4 to 22.8]	< 0.001
≥ 80 years	194 (3.3)	84.8	–	–	–	–	–	–	17.7*	[13.4 to 22.1]	< 0.001

B) Temporal trend of hospitalizations due to malignant neoplasm of the female breast in the state and in the 10 municipalities of Alagoas that presented the highest rates of this variable											
			Trend 1			Trend 2			Total period		
State/Municipality	Number of hospitalizations (%)	Hospitalization rate over the period/100,000 inhabitants	Period	APC	95% CI	Period	APC	95% CI	AAPC	95% CI	P-value
State of Alagoas	5801 (100)	36.1	–	–	–	–	–	–	14.0*	[12.3 to 15.8]	< 0.001
Arapiraca	737 (12.7)	65.7	–	–	–	–	–	–	13.3*	[10.6 to 16.1]	< 0.001
Major Isidoro	48 (0.8)	49.9	–	–	–	–	–	–	15.8*	[6.2 to 26.2]	< 0.001
Maceió	2444 (42.1)	49.3	–	–	–	–	–	–	12.7*	[9.8 to 15.6]	< 0.001
Jaramataia	13 (0.2)	47.2	–	–	–	–	–	–	137.7*	[44.4 to 291.3]	< 0.001
Barra de São Miguel	17 (0.3)	44.6	–	–	–	–	–	–	160.7*	[32.2 to 413.9]	< 0.001
São Miguel dos Milagres	16 (0.3)	44.4	–	–	–	–	–	–	-15.8	[-59.3 to 74.4]	0.6
Olho d’Água Grande	11 (0.2)	44.1	–	–	–	–	–	–	211.4*	[105.1 to 372.8]	< 0.001
Palmeira dos Índios	162 (2.8)	44.0	2009-2011	128.3	[-22.9 to 576.1]	2011-2019	4.4	[-0.9 to 9.9]	22.1*	[2.3 to 45.7]	< 0.001
Barra de Santo Antônio	31 (0.5)	43.3	–	–	–	–	–	–	42.2	[-0.1 to 102.4]	0.1
Paulo Jacinto	16 (0.3)	42.2	–	–	–	–	–	–	151.0*	[31.3 to 379.9]	< 0.001

No joinpoint was observed in this indicator; *Statistically significant P < 0.05. APC = annual percentage change; CI = confidence interval; AAPC: average annual percentage change.

We observed that most hospitalizations due to breast cancer (60.2%) occurred in 10 of the 102 municipalities of Alagoas, especially in Maceió (n = 2,440), Arapiraca (n = 737) and Palmeira dos Índios (n = 162); and, in relation to the average rate over the period, in Arapiraca (65.7), Major Isidoro (49.9) and Maceió (49.3). Among these municipalities, eight presented an increasing trend, especially the municipality of Olho d’Água Grande (AAPC = 211.4; 95% CI = 105.1 to 372.8; P-value < 0.001). Barra de Santo Antônio and São Miguel dos Milagres showed parallel stationary temporal behavior (Table 1B).

**Figure 2.**
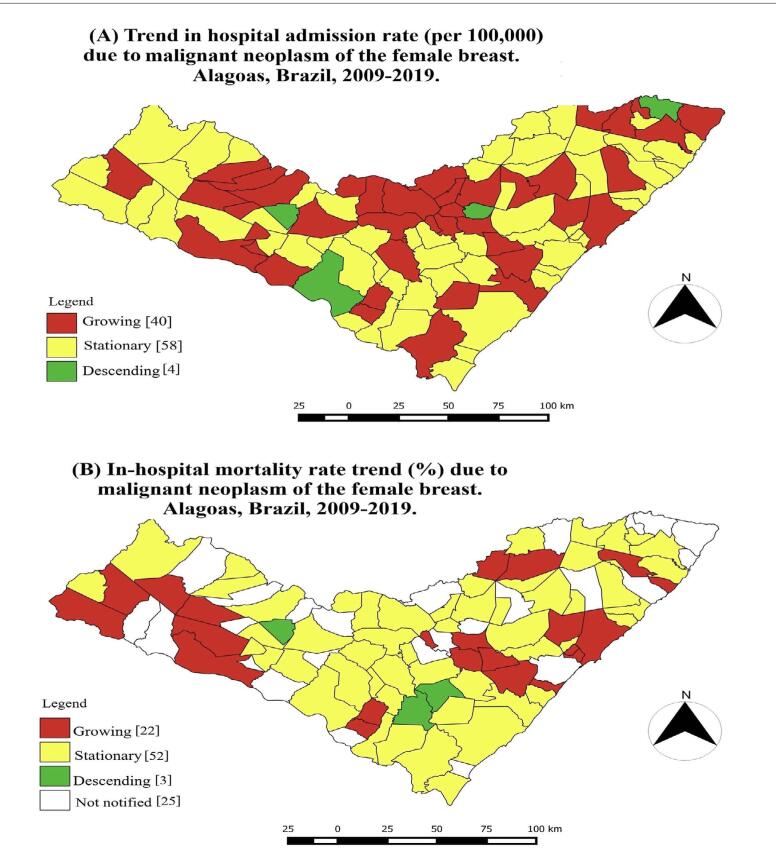
Distribution of the trends in hospital admission rate (per 100,000 inhabitants) and in-hospital mortality rate (%) due to female breast cancer in the municipalities of the state of Alagoas in the period from 2009 to 2019. Brazil, 2021.

Among all the 102 municipalities in Alagoas, there was an increasing trend of hospitalizations in 39.2% (n = 40), particularly in Monteirópolis (AAPC = 212.8; 95% CI = 124.2 to 336.3; P-value < 0.001), Belo Monte (AAPC = 212.2; 95% CI = 212.2 to 212.2; P-value < 0.001) and Olho d’Água Grande (AAPC = 211.4; 95% CI = 105, 1 to 372.8; P-value < 0.001), which provided the three largest percentage changes over the period (Figure 2A).

Four municipalities (3.9%) showed decreasing trends of hospitalizations. These were Olivença (AAPC = -50.6; 95% CI = - 70.5 to -17.3; P-value < 0.001), Jacuípe (AAPC = -43.2; 95% CI = - 57.2 to -24.7; P-value < 0.001), Pindoba (AAPC = -25.8; 95% CI = - 25.8 to -25.8; P-value < 0.001) and Traipu (AAPC = -17.6; 95% CI = -28.6 to -5.0; P-value < 0.001), which are located, respectively, in the metropolitan areas of Médio Sertão, Zona da Mata, Vale do Paraíba and Agreste (Figure 2A).

### In-hospital mortality analysis

Regarding age groups, 31.1% of hospital deaths (n = 197) occurred among women between 50 and 59 years old. Among people under 20 years old, no deaths were recorded. In the age groups of 20-29, 40-49, 50-59, 60-69 and 70-79 years, there was a tendency towards stability. The age group from 30 to 39 years old showed a growth trend between 2009 and 2011, and from 2011 onwards, it showed a pattern of stability. In considering the complete time series, growth was observed (AAPC = 58.9; 95% CI = 12.1 to 125.2; P-value < 0.001) (Table 2A).

**Table 2.** Temporal trend of in-hospital mortality due to malignant neoplasm of the female breast in the state of Alagoas and in the 10 municipalities with the highest rates of this variable in the period from 2009 to 2019 and according to the age group. Brazil, 2021

A) Temporal trend of in-hospital mortality due to malignant neoplasm of the female breast according to age group														
			Trend 1			Trend 2			Trend 3			Total Period		
Age group	Number of hospital deaths (%)	In-hospital mortality rate over the period (%)	Period	APC	95% CI	Period	APC	95% CI	Period	APC	95% CI	AAPC	95% CI	P-value
> 20 years	0													
20-29 years	8 (1.3)	6.1	–	–	–	–	–	–	–	–	–	40.4	[-35.9 to 207.6]	0.4
30-39 years	59 (9.3)	9.8	2009-2011	1204.9*	[57.5 to 10712.6]	2011-2019	-6.2	[-17.5 to 6.8]	–	–	–	58.9*	[12.1 to 125.2]	< 0.001
40-49 years	138 (21.8)	10.8	2009-2012	29.5*	[6.1 to 58.0]	2012-2019	-4.6*	[-8.8 to -0.3]	–	–	–	4.5	[-1.0 to 10.3]	0.1
50-59 years	197 (31.1)	14.6	–	–	–	–	–	–	–	–	–	1.7	[-2.4 to 6.0]	0.4
60-69 years	126 (19.9)	13.7	–	–	–	–	–	–	–	–	–	4.9	[-1.5 to 11.8]	0.1
70-79 years	70 (11.1)	16.0	–	–	–	–	–	–	–	–	–	-3.2	[-7.8 to 1.6]	0.2
≥ 80 years	35 (5.5)	17.7	2009-2011	9522.3	[-52.1 to 1930910.6]	2011-2019	0.5	[-26.4 to 37.3]	–	–	–	150.2*	[4.6 to 498.8]	< 0.001

B) Temporal trend of in-hospital mortality due to malignant neoplasm of the female breast in the state and in the 10 municipalities that presented the highest rates of this variable														
			Trend 1			Trend 2			Trend 3			Total Period		
State/Municipality	Number of hospital deaths (%)	In-hospital mortality rate over the period (%)	Period	APC	95% CI	Period	APC	95% CI	Period	APC	95% CI	AAPC	95% CI	P-value
State of Alagoas	633 (100)	11.7	2009-2012	24.6*	[5.7 to 46.8]	2012-2019	-2.4	[-5.7 to 1.1]	–	–	–	5.0*	[0.5 to 9.7]	< 0.001
Porto Calvo	6 (0.9)	29.3	–	–	–	–	–	–	–	–	–	48.3	[-26.7 to 200.1]	0.2
Igreja Nova	3 (0.5)	25.0	–	–	–	–	–	–	–	–	–	-8.4	[-57.8 to 98.7]	0.8
Cajueiro	4 (0.6)	22.1	–	–	–	–	–	–	–	–	–	37.9	[-25.8 to 156.0]	0.3
Pilar	10 (1.6)	21.7	2009-2011	11940.0	[-94.7 to 27301204.7]	2011-2016	25.1	[-72.1 to 462.1]	2016-2019	-95.9*	[-99.7 to -33.6]	11.9	[-65.5 to 262.9]	0.9
Lagoa da Canoa	5 (0.8)	21.7	–	–	–	–	–	–	–	–	–	-38.7	[-71.5 to 31.9]	0.2
Jequiá da Praia	2 (0.3)	20.0	–	–	–	–	–	–	–	–	–	0.0	[-44.6 to 80.4]	1.0
Colônia Leopoldina	2 (0.3)	20.0	–	–	–	–	–	–	–	–	–	0.0	[-46.6 to 87.3]	1.0
Estrela de Alagoas	4 (0.6)	18.7	–	–	–	–	–	–	–	–	–	-7.4	[-47.9 to 64.7]	0.8
Batalha	3 (0.5)	17.5	–	–	–	–	–	–	–	–	–	-4.1	[-59.0 to 124.3]	0.9
Atalaia	9 (1.4)	16.5	–	–	–	–	–	–	–	–	–	34.8	[-39.0 to 198.0]	0.4

No joinpoint was observed in this indicator; *Statistically significant P < 0.05. APC = annual percentage change; CI = confidence interval; AAPC: average annual percentage change.

In the age group from 40 to 49 years, there was a temporal inflection in 2012, going from a growth trend (AAPC = 29.5; 95% CI = 6.1 to 58.0; P-value < 0.001) to a trend of decline (AAPC = -4.6; 95% CI = - 8.8 to -0.3; P-value < 0.001). The age group ≥ 80 years showed a linear growth trend (AAPC = 150.2; 95% CI = 4.6 to 498.8; P-value < 0.001) (Table 2A).

In addition, 28.6% (n = 22) of the 77 municipalities showed an increasing trend, particularly Pão de Açúcar (AAPC = 325.6; 95% CI = 325.6 to 325.6; P-value < 0.001), Inhapi (AAPC = 244.1; 95% CI = 101.6 to 487.2; P-value < 0.001) and Belém (AAPC = 150.8; 95% CI = 69.7 to 270.7; P-value < 0.001). There was a decreasing trend in 3.9% (n = 3) of the municipalities: these were Junqueiro (AAPC = -44.9; 95% CI = - 68.8 to -2.7; P-value < 0.001), São Sebastião (AAPC = -40.6; 95% CI = - 56.5 to -18.8; P-value = 0.2) and Olivença (AAPC = -37.0; 95% CI = - 59.0 to -3.0; P-value < 0.001). The first two of these are located in the metropolitan region of Agreste and the last in Médio Sertão (Figure 2B).

A total of 77 municipalities registered deaths. The municipalities with the highest numbers of deaths were Maceió (n = 282; 11.8%), Arapiraca (n = 71; 12.3%) and Rio Largo (n = 23; 14.0%). The ten municipalities with the highest rates accounted for only 7.5% (n = 48) of total deaths, and all these municipalities presented stationary behavior (Table 2B).

## DISCUSSION

This study identified the temporal trend of mortality (per 100,000 inhabitants) and in-hospital mortality rate (%) due to female breast cancer in the state of Alagoas. Within these rates, variations between the municipalities of the state were observed. In 2019, data from Brazil, regarding the trend of breast cancer incidence rates according to age groups, showed that the highest rates started from 70 years old, while there was a decreasing trend for the 40 to 49-year age group and stability for the 20 to 39 and 50 to 69-year age groups.^13^ Those results are in line with ours, since the values were similar to those found in the present study. In Alagoas, age groups over 30 years old showed an upward trend, especially the group between 70 and 79 years old. The higher rates for age groups of 70 years and over were consistent with what has been observed worldwide, given that the risk of developing cancer increases with advancing age.^14^

It is well known that the aging process is the main risk factor for breast cancer. Thus, this has become one of the biggest challenges in public health. In this physiological process, the decline of individuals’ immune system and dysregulation of the neuroendocrine system make their organisms more vulnerable to infections and other pathological processes.^15,16^ In addition, accumulation of mutations in the DNA of cells over individuals’ lives is the reason why cancer occurs mostly in the elderly.^16^ This could explain the higher rates of both hospital admissions and deaths due to breast cancer among women from the perimenopause onwards.

Another plausible explanation for the greater numbers of findings of cancer among older people is that there is a tendency to perform tests according to advancement of age. Thus, older people are vulnerable to discovery of cancers at more advanced stages. This consequently makes them subject to more radical surgical treatments, which reduces the opportunity for cure and increases the chances of complications and longer hospital stay,^17^ thereby facilitating progression to death.

In addition to these factors, another physiological factor that may predispose women to breast cancer is the changes that they undergo during puberty. This phase of life involves increases in the serum levels of estrogen and prolactin, which have major effects on the mammary epithelium. In a survey conducted in the city of Cuiabá, Mato Grosso, Brazil, among 19 women, it was observed that 47.4% of them presented menarche in the age group between 10 and 13 years old, at an average age of 13.31. It was therefore suggested that early menarche and onset of a regular ovulatory cycle would increase the risk of breast cancer, since estrogen levels are higher during the normal luteal phase, and the cumulative estrogen exposure index is higher.^18,19^

Regarding in-hospital mortality, in 2012 there was an interruption of the growth trend in Alagoas and the age group of 40-49 years shifted from an increasing trend to a declining trend. It is likely that the implementation of the *Consensus for Breast Cancer Control,* prepared by the Brazilian Health Ministry in 2004 may have influenced this result.^20^ This document recommends that clinical examination of the breasts should be performed annually from the age of 40 years onwards and that mammographic screening should be performed every two years from 50 to 69 years of age. Among high-risk women, annual clinical and mammographic examinations are recommended from the age of 35 years onwards. Thus, if diagnosis and treatment are performed early, the prognosis is considered good, with reduced mortality. In this scenario, the result is decreased mortality rates.^20^

Although the state capital of Alagoas (Maceió) had the highest number of hospital deaths due to breast cancer among women, it had a lower in-hospital mortality rate than other municipalities in inland areas. Among the state capitals of the northeastern region of Brazil, mammography coverage estimates have ranged from 64.3% to 84.4%.^21^ In municipalities away from the state capitals, mortality due to breast cancer has increased in all regions since the 1990s.^22^ It is possible that among women in non-metropolitan regions, access to mammography is lower than among residents in the state capitals. This was shown in a study conducted in 2015, in which living in an urban area increased the likelihood of undergoing a mammographic examination by 10.97 percentage points^23^ and therefore increased the likelihood of early detection of cancer.

One possible reason for this disparity may arise from differences in women’s exposure to risk factors and diagnostic practices. It is noteworthy that breast cancer tends to be diagnosed at more advanced stages in less developed regions^24^ in which access to the healthcare system is lower. Moreover, it is known that mortality rates show a correlation with access to healthcare services and the quality of care that is offered to women with breast cancer.^13^ In a study published in 2017, it was seen that a proportion of the preventable deaths from breast cancer was correlated with unequal access to treatment between the rich and needy populations, and that this disparity is quite common in developing countries, such as Brazil.^25^

Another study also showed that the highest mortality rates were found in municipalities with more than 500,000 inhabitants and in those with populations of up to 5,000 inhabitants.^26^ These findings corroborate what was found in our analyses when we addressed the smaller municipalities. The municipalities that presented high rates of in-hospital mortality, according to the 2010 census, had small populations. It can be suggested that in these places there is a greater possibility of shorter reach of preventive actions, as well as a lack of infrastructure, which can hinder the diagnosis and early treatment of the disease. This would form a possible explanation for the findings. In large cities, however, the high mortality rate may reflect the fact that women with breast cancer may move to large centers in search of better treatments, along with urbanization and possible changes in reproductive patterns.

Therefore, the need to promote broader access to cancer diagnoses in Brazil can be stressed, with special attention to women with lower socioeconomic status. In particular, attention is required for women residing in inland municipalities in Alagoas, which is the scenario of the present study. It has been noted that there is uneven distribution of the numbers of mammography devices in the state of Alagoas, in which more than 93.3% (n = 14) are concentrated in the two most populous municipalities: Maceió, with 53.3% (n = 8), and Arapiraca (40.0%, n = 6).^27^

Over time, the number of hospital admissions in the state of Alagoas due to breast cancer has grown. This trend corroborates studies that analyzed the increased incidence and mortality due to this type of cancer in Brazil.^13,28^ In addition, there were increases in the rate of hospital admissions over the period, in 41.2% (n = 42) of the 102 municipalities in the state. This gave rise to a broad sample for regional characterization of this category of cancer. In the panorama of hospitalizations in the state, the rates of hospital admissions were concentrated among patients who were residents of 10 municipalities and among these, three cities (Arapiraca, Maceió and Palmeira dos Índios) stood out. These three cities had the highest numbers of hospital admission records. It can be inferred that, given that these are the most populous cities in the state,^11^ there is a greater concentration of provision of hospital resources. This relationship is similar to what was seen in a study carried out in Bahia on hospitalizations due to malignant breast cancer.^29^ It can also be deduced that some women who had been admitted to hospitals in these more populous cities stayed in these locations and provided addresses in these cities as their home address.

It needs to be mentioned that this study had some limitations that should be taken into account: i) the secondary data on hospital admissions reflected the hospital flow, and so it is possible that the same person was hospitalized more than once and, consequently, the number of admissions may have exceeded the number of people hospitalized; ii) cancer patients may not need hospitalization and, therefore, the number of hospitalizations may have been underestimated in relation to the total number of people with cancer in our population; iii) the scarcity of studies in this area may have made comparative analysis difficult; and iv) the database used only took public hospitals into consideration.

## CONCLUSION

There was a higher percentage of hospital admissions and deaths due to breast cancer in the state of Alagoas among women aged 50 to 59 years, considering the period from 2009 to 2019. Over this period, according to age group, all age ranges showed an increasing trend for hospitalizations, with the exception of women under 20 years old, for whom hospitalization decreased, and those between 20 and 29 years, whose rate remained stable. Regarding in-hospital mortality, all ranges showed a stable pattern, with the exception of 30 to 39 and ≥ 80 years, which increased.

There were growing trends of hospital admissions and in-hospital mortality in the state. Among the different localities in Alagoas, most of them showed a stationary trend, followed by an increasing trend in both hospitalization and mortality rates.

In this context, knowing the profile of hospital admissions due to breast cancer makes it possible to understand the temporal panorama with more reliability and with its local specificities. Thus, this promotes regional indicators that provide data for actions aimed at this portion of the population. In addition, it is possible to measure the quality and equity of access to these oncology services and to identify the areas of greater social vulnerability.
